# The epidemiology and outcomes of prolonged trauma care (EpiC) study: methodology of a prospective multicenter observational study in the Western Cape of South Africa

**DOI:** 10.1186/s13049-022-01041-1

**Published:** 2022-10-17

**Authors:** Krithika Suresh, Julia M. Dixon, Chandni Patel, Brenda Beaty, Deborah J. del Junco, Shaheem de Vries, Hendrick J. Lategan, Elmin Steyn, Janette Verster, Steven G. Schauer, Tyson E. Becker, Cord Cunningham, Sean Keenan, Ernest E. Moore, Lee A. Wallis, Navneet Baidwan, Bailey K. Fosdick, Adit A. Ginde, Vikhyat S. Bebarta, Nee-Kofi Mould-Millman

**Affiliations:** 1 Department of Biostatistics and Informatics, Colorado School of Public Health, University of Colorado, Aurora, CO USA; 2 Department of Emergency Medicine, School of Medicine, University of Colorado, 12631 E. 17th Ave, Room 2612, MS C326, Aurora, CO 80045 USA; 3 Adult and Child Consortium for Health Outcomes Research and Delivery Science (ACCORDS), University of Colorado Anschutz Medical Campus, Aurora, CO USA; 4 Department of Pediatrics, McGovern Medical School, University of Texas Health Science Center at Houston, Houston, TX USA; 5 Emergency Medical Services, Western Cape Government Health, Cape Town, South Africa; 6 Department of Surgery, Faculty of Medicine and Health Sciences, Stellenbosch University, Cape Town, South Africa; 7 Division of Forensic Medicine, Department of Pathology, Faculty of Medicine and Health Sciences, Stellenbosch University, Cape Town, South Africa; 8 U.S. Army Institute of Surgical Research, San Antonio Medical Center, San Antonio, TX USA; 9 Brooke Army Medical Center, Fort Sam Houston, San Antonio, TX USA; 10 Joint Trauma System, Defense Health Agency, Fort Sam Houston, San Antonio, TX USA; 11 Department of Emergency Medicine, The Center for COMBAT Research, School of Medicine, University of Colorado, Aurora, CO USA; 12 Ernest E Moore Shock Trauma Center, Denver Health and Hospital Authority, Denver, CO USA; 13 Division of Emergency Medicine, University of Cape Town, Cape Town, South Africa

**Keywords:** Emergency care system, Epidemiology, Emergency medical services, Global health, Military, Mortality, Morbidity, Prolonged duration until care, Trauma database

## Abstract

**Background:**

Deaths due to injuries exceed 4.4 million annually, with over 90% occurring in low-and middle-income countries. A key contributor to high trauma mortality is prolonged trauma-to-treatment time. Earlier receipt of medical care following an injury is critical to better patient outcomes. Trauma epidemiological studies can identify gaps and opportunities to help strengthen emergency care systems globally, especially in lower income countries, and among military personnel wounded in combat. This paper describes the methodology of the “Epidemiology and Outcomes of Prolonged Trauma Care (EpiC)” study, which aims to investigate how the delivery of resuscitative interventions and their timeliness impacts the morbidity and mortality outcomes of patients with critical injuries in South Africa.

**Methods:**

The EpiC study is a prospective, multicenter cohort study that will be implemented over a 6-year period in the Western Cape, South Africa. Data collected will link pre- and in-hospital care with mortuary reports through standardized clinical chart abstraction and will provide longitudinal documentation of the patient’s clinical course after injury. The study will enroll an anticipated sample of 14,400 injured adults. Survival and regression analysis will be used to assess the effects of critical early resuscitative interventions (airway, breathing, circulatory, and neurologic) and trauma-to-treatment time on the primary 7-day mortality outcome and secondary mortality (24-h, 30-day) and morbidity outcomes (need for operative interventions, secondary infections, and organ failure).

**Discussion:**

This study is the first effort in the Western Cape of South Africa to build a standardized, high-quality, multicenter epidemiologic trauma dataset that links pre- and in-hospital care with mortuary data. In high-income countries and the U.S. military, the introduction of trauma databases and registries has led to interventions that significantly reduce post-injury death and disability. The EpiC study will describe epidemiology trends over time, and it will enable assessments of how trauma care and system processes directly impact trauma outcomes to ultimately improve the overall emergency care system.

*Trial Registration*: Not applicable as this study is not a clinical trial.

## Background

### The global burden of injury

Intentional and unintentional injuries are a leading cause of mortality worldwide, claiming over 4.4-million lives annually [1]. The World Health Organization has ranked motor vehicle collisions, falls, and interpersonal violence within the top 20 global causes of death and disability [1, 2]. Further, a large proportion of trauma deaths occur prior to arrival at a hospital [3, 4]. To significantly reduce global trauma mortality, there is an immediate need to improve patient-level outcomes in prehospital and in-hospital settings, in concurrence with prevention efforts. Prehospital care (e.g., basic care in ambulances) is especially critical in averting poor outcomes at the population level because it represents the earliest formal opportunity for the emergency care system to initiate life-saving care [5].

Trauma disproportionately impacts populations in low-and middle-income countries (LMICs) with over 90% of global injury-related deaths occurring in these populations [6, 7]. Individuals residing in low-resource settings experience twice the trauma mortality of high-resource settings, partly due to poor access and delays in receiving critical resuscitative interventions (CRI) and definitive trauma care [8–12]. South Africa, specifically, has a transportation-related fatality rate (39.7 per 100,000) that is twice the global average and mortality from interpersonal violence that is among the highest in the world, both of which are partially due to prolonged duration from time of injury to definitive care [11]. There is an urgent need to improve emergency care systems in South Africa via comprehensive trauma epidemiological studies. These population-based studies that collect individual patient-level data can inform clinical care and policies to maximally impact populations, thus making them an essential first step to reducing the burden of trauma [6, 13].

### The burden of trauma in U.S. military populations

In addition to civilian populations, trauma is a major cause of morbidity and mortality amongst military populations. Battlefield hemorrhage, for example, is the leading cause of fatalities with potentially survivable injuries amongst U.S. military personnel [14, 15]. Yet only one-in-four combat fatalities is estimated to have potentially survivable injuries, and outcomes depend on the timely delivery of early prehospital CRIs [4]. Several urban, U.S.-based civilian prehospital trauma studies have been conducted in relatively small samples of patients and report relatively short transport times to definitive care [16, 17], thus generalizability and relevance to the battlefield are limited. The trauma profile of the Western Cape in South Africa, due to gang “wars” and interpersonal firearm violence, for example, better reflects the military paradigm of severe injury patterns, health system resource limitations, and prolonged trauma-to-treatment time [18]. Thus, epidemiologic studies conducted in this region can provide important findings for use in military triage and transport decisions.

### The impact of delayed care on morbidity and mortality

To yield the best outcomes after critical injuries in both civilian and military settings, patients should receive prompt treatments at the point of injury and rapid transport directly to a capable trauma center [4, 19, 20]. Delays experienced between the time of injury to arrival at a surgically capable trauma facility negatively impact outcomes for patients with severe injury [21]. In civilian contexts worldwide, delays are predominantly due to slow prehospital response or transport, or lengthy times spent in an initial non-trauma center. In U.S. urban and rural areas, studies have shown an inverse correlation between prehospital time and survival in motor vehicle victims [22]. Moreover, those in U.S. rural areas are 50% more likely to die from all-cause trauma compared to their urban counterparts because of longer prehospital transport times [22]. Studies examining the effects of prehospital time on mortality are sparse, with even fewer conducted in LMICs [9, 19, 23–27]. Within the U.S. military’s Africa Command area of operations, transportation times are frequently prolonged, which potentially worsens trauma outcomes [28–30]. To improve survival in battlefield situations where the duration of prehospital casualty care is prolonged, military trauma experts have called for scientific evidence to better inform clinical guidelines [31].

### The epidemiology and outcomes of prolonged trauma care (EpiC) study

To address scientific gaps in both global civilian public health and military medicine, the United States Department of Defense (DOD) funded the study “Epidemiology and Outcomes of Prolonged Trauma Care (EpiC): A Multicenter Prehospital Observational Study in the Western Cape of South Africa” [32, 33]. EpiC aims to advance the understanding of epidemiology and outcomes of trauma and will guide future interventional research. This will be the first multi-institutional trauma database to be implemented in South Africa that prospectively collects trauma case data from point of injury to outcome, linking pre- and in-hospital care with mortuary data. EpiC will inform public health officials and key stakeholders with evidence on how trauma treatments and system processes directly impact changes in patient outcomes, including trends in trauma epidemiology and care over time. Collecting longitudinal data with key time stamps from the public emergency care system across multiple regions and facility types will allow for comprehensive comparisons between patient sub-populations and outcomes [6, 34]. The EpiC study will determine how delivery of critical resuscitative interventions and the timeliness of these interventions impacts the morbidity and mortality of civilian patients in the Western Cape who experience significant trauma.

## Methods and design

### Objectives

The specific objectives of the EpiC study are:

1. To assess the effect of delivery and timeliness of critical resuscitative interventions (CRI) on the primary 7-day mortality outcome and secondary mortality (24-h, 30-day) and morbidity outcomes (need for operative interventions, secondary infections, and organ failure).
2. To assess the effect of individual resuscitative interventions (airway, breathing, circulatory, and neurologic) on similar mortality and morbidity outcomes.

We hypothesize that failure to deliver CRIs and longer durations of trauma-to-CRI time will be associated with increased risk of mortality and worse morbidity outcomes.

### Study setting

**Fig. 1:**
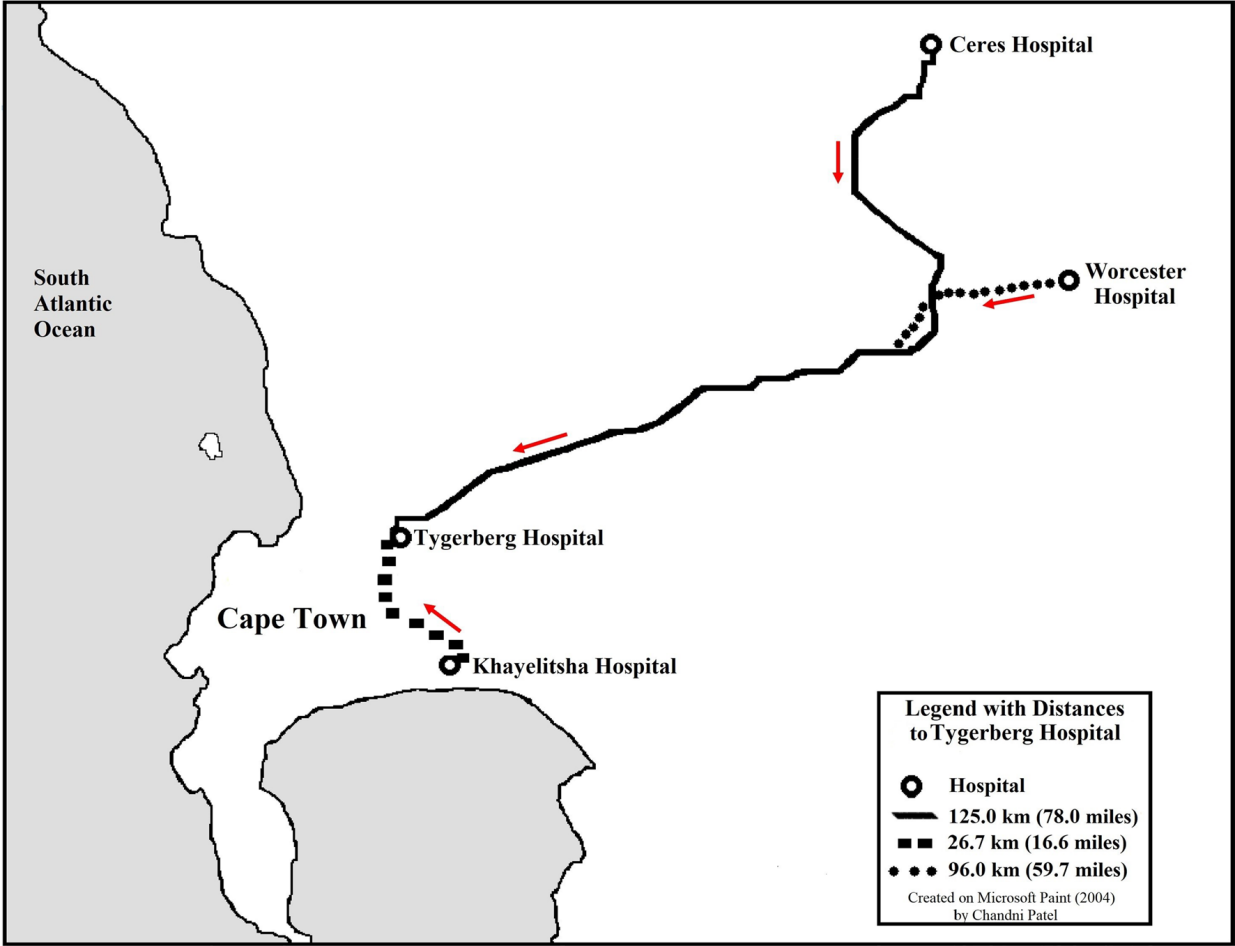
Relative transport distances from EpiC study sites to the tertiary trauma care center (Tygerberg Hospital)

South Africa is an upper-middle income country with disparate trauma outcomes based on sex, socio-economic status, unemployment, urbanization, and race [11]. A call for urgent action by numerous public health and subject matter experts has been made to improve emergency care systems, both in South Africa as a whole, and in the Western Cape specifically [13]. EpiC will be implemented in a cross-section of the Western Cape emergency care system, in government-operated institutions and agencies. Study sites will include two district hospitals, one regional hospital, and their referral trauma center. EpiC will also include four Western Cape Government Emergency Medical Services (WCG EMS) ambulance bases and two forensic pathology laboratories that serve all four hospitals (Table 1, Fig. 1).

**Table 1:** Characteristics of EpiC study sites

Institution/Agency	Level of care/Facility type	Number of sites	Community served	Distance to trauma center (km)	Number of hospital beds
Tygerberg	Tertiary trauma center	1	Urban	0	1384
Khayelitsha	District hospital	1	Urban	26.7	230
Ceres	District hospital	1	Rural	125.5	28
Worcester	Regional hospital	1	Rural	96.0	277
WCG EMS	Government ambulance	4	Urban, Rural	n/a	n/a
Forensic Pathology Service (FPS)	Government pathology service	2	Urban, Rural	n/a	n/a

n/a = not applicable; WCG EMS = Western Cape Government Emergency Medical Services

Tygerberg Hospital is the tertiary trauma (referral) center that provides specialty surgical and trauma care to a catchment area of 3.4 million people and attends to over 107,000 admissions annually of which 12,000–15,000 are seriously injured persons [35, 36]. All other EpiC hospital sites (Khayelitsha, Ceres, and Worcester Hospitals) refer complex and critical trauma patients to Tygerberg Hospital for a full range of trauma subspecialty care and consultations [37]. Khayelitsha Hospital is a frontline, district level facility that serves a dense urban catchment area of over 400,000 people and treats around 3,000 patients per month in the Emergency Center, many with injuries from interpersonal violence and motor vehicle collisions [38–40]. Worcester Hospital is a regional level facility that serves a rural population of 917,000 and can provide intensive care and general surgical care. Ceres Hospital is a rural, small district level facility without specialist physicians and offers basic trauma care. Patients at Ceres Hospital who require sub-specialty trauma and surgical care are stabilized and transferred to a trauma center by WCG EMS ambulances.

The WCG EMS is a public prehospital agency that annually treats and transports over 500,000 patients to hospitals, 40% due to trauma [41, 42]. Most ambulances are staffed by basic and intermediate life support providers, whereas advanced life support capable crews are reserved for the most critically injured patients [41, 42]. EpiC will include ambulances servicing catchment areas of the four participating hospitals. The Forensic Pathology Service (FPS) of the Western Cape has a constitutional mandate to perform autopsies on all unnatural deaths, including all trauma deaths. Two FPS labs will participate in this study: the Tygerberg FPS lab (includes deaths of patients from Tygerberg and Khayelitsha Hospital) and the Worcester FPS Lab (includes Worcester and Ceres Hospital deaths) [43].

### Study design

This is a prospective, multicenter cohort study of trauma patients that will be conducted over a 6-year period, divided into 3 phases: a preparatory Phase 1 (year 1), a pilot implementation Phase 2 (year 2), and a main study Phase 3 (years 3–6).

***Phase 1. ***During the preparatory phase, we will create an evidence-based, expert-consensus data dictionary and data capture tools. This process will involve a review and adaptation of five international high-quality trauma data dictionaries (DOD Trauma Registry [44], National Emergency Medical Services Information System [45], National Trauma Data Standard [46], Pan-Asian Trauma Outcomes Study [47], and World Health Organization dataset for injury [48]), a scoping review of literature published in the past 10 years [49], and a modified Delphi consensus-building process using a panel of 20 military and civilian trauma clinical and research experts.

***Phase 2. ***We will conduct a pilot study at all study sites to assess the feasibility of implementation, data collection, and analysis. During this phase we will enroll ~ 1,000 patients with moderate and high acuity trauma (using the South Africa Triage Scale [SATS] acuity score), assess the frequency of erroneous and missing data, and conduct preliminary data analyses to describe the study cohort [50].

***Phase 3.*** In the main study, we will prospectively collect data from all study sites and perform statistical analyses to answer the research objectives.

### Inclusion and exclusion criteria

The EpiC study will include adult patients aged 18 years and older with a clinical encounter for a traumatic injury at one or more of our 10 study sites. Eligible patients will meet one of the following criteria: (1) arrive and/or depart by WCG EMS to/from a participating site, (2) transported via WCG EMS with initial signs of life or receive prehospital resuscitation care, but die in the ambulance prior to hospital arrival, (3) were alive at the time of the EMS call but were deceased upon their arrival, or (4) emergency center walk-ins. Those under the age of 18 years, prisoners, injury onset > 24-h prior to arrival at first study site, bites, stings, other forms of envenomation, toxicologic injuries, drownings, patients who are found deceased on scene, and/or patients transported via private EMS (non-WCG EMS) will be excluded.

### Participant recruitment and informed consent

Eligible patients will be identified daily at all study sites. The clinical records of patients meeting inclusion criteria will be reviewed and data will be abstracted into the study database. Data for each patient will be linked as they traverse the emergency care system. Consistent with other observational emergency care studies, a waiver of informed consent was approved by the ethics boards to practically carry out this minimal risk research on patients with emergent (i.e., time-sensitive, and life-threatening) conditions.

### Variables, outcomes, and study measures

Epidemiologic information will be collected for each study participant for their entire clinical course at all associated study sites, including: patient demographic information (e.g., age, sex, race, and comorbidities); injury details (e.g., time, type, and intent); facility data (e.g., hospital name, trauma level, clinician qualifications, units of care); clinical data (e.g., vital signs, medications administered, critical interventions, operations performed); and, trauma system process (e.g., key dates and times [51]). Table 2 describes the exposures, predictors, and outcomes of interest in the EpiC study.

**Table 2:** Key EpiC Study Exposure, Predictors, and Outcome Measures

Exposures	Definition	Variable Type	Data Source
1^O—^Critical resuscitative interventions (CRI)	Prehospital and in-hospital resuscitative interventions or therapeutics, delivered for the patient’s traumatic injury (e.g., thoracostomy for tension pneumothorax), within the first 24 h post-injury	Binary	EMS and/or Hospital
1^O^—Trauma-to-CRI time	Duration of time from injury to receiving a critical resuscitative intervention for the dominant injury	Continuous	EMS, Hospital, and/or Pathology

Predictors	Definition	Variable Type	Data Source
Age	Span of years lived at the time of injury	Continuous	EMS, Hospital, or Pathology
Sex	Biological sex given at birth	Categorical	EMS, Hospital, or Pathology
Comorbidities	One or more pre-existing disease or conditions present in the patient that are trauma-relevant chronic conditions (e.g., bleeding disorder)	Binary	EMS or Hospital
Socioeconomic status	Based on hospital insurance status	Categorical	Hospital
Mechanism of injury	Main force that created the injury or how injury was inflicted (e.g., firearm, Struck/hit, stabbing or cut, vehicular injury, fall, thermal, choking/hanging, iatrogenic, other)	Categorical	EMS, Hospital, or Pathology
Provider level	Health care provider qualifications (e.g., rank of EMS provider, type of doctor)	Categorical	EMS
Injury severity	ISS calculated as the sum of squares of 3 worst AIS scores. Low (< 12) or high (≥ 12)	Continuous, Binary	EMS, Hospital, or Pathology

Outcomes	Definition	Variable Type	Data Source
1^O—^Ambulance death or 7-day in-hospital mortality	All-cause mortality in the ambulance or within the hospital in the first 7-days post-injury	Time-to-event	EMS, Hospital, or Pathology
2^O^—30-day in-hospital mortality	All-cause mortality at any point during the hospital stay	Time-to-event	Hospital or Pathology
2^O^—Non-resuscitative surgical interventions	Post-injury interventional procedures such as fasciotomies, limb amputation, and laparotomies	Binary	Hospital
2^O^—Secondary infections	Post-injury infections such as wound infections, pneumonia, and sepsis	Binary	Hospital
2^O^—Organ failure	Organ failure will be assessed by individual organ (acute kidney injury, coagulopathy, and acute lung injury) and by a composite multiple organ failure score such the SOFA score up to 7-days	Continuous	Hospital

1^O^ = primary exposure or outcome; 2^O^ = secondary exposure or outcome; Data Source: EMS data is abstracted from WCG EMS Electronic Patient Care Reports, hospital data is abstracted from handwritten medical charts, and pathology data is abstracted from handwritten postmortem/autopsy reports from the Forensic Pathology Service of South Africa. EMS = Emergency Medical Services, ISS = Injury Severity Score, AIS = Abbreviated Injury Scale (anatomical score), SOFA = Sequential Organ Failure Assessment

The primary outcome of mortality will be defined as time to ambulance death or in-hospital death within 7 days of injury. The definition of examining mortality within 7 days was previously selected by a 20-person expert trauma panel to be the ideal duration to assess deaths for a study focused on the effects of early trauma resuscitation [52]. A study of three trauma-focused randomized controlled trials in urban U.S. settings have demonstrated that most hemorrhagic deaths occur in hospitals and within 6 h of patient encounter [51]. However, in our setting we examine a longer window since early deaths are more likely to occur at the scene due to the higher prevalence of penetrating injuries and fewer ambulance resources, and consequently later deaths will likely occur post-EMS transfer and hospital arrival. We will define the secondary outcomes of 24-h and 30-day mortality as time to in-ambulance or in-hospital mortality from injury. Other secondary outcomes include patients’ morbidity and need for operative interventions (those that are not considered damage control surgery), secondary infections (including wound infections, pneumonia, and sepsis), and organ failure (defined with the Sequential Organ Failure Assessment (SOFA) Score [53, 54]).

The primary exposures of interest are the delivery of CRI and trauma-to-CRI time. The delivery of CRIs will be defined as the performance of a resuscitative intervention (airway, breathing, circulatory, or neurologic) within the first 24-h from the time of injury. The trauma-to-CRI time will be defined as the duration from experiencing the injury to receiving a critical resuscitative intervention, which could occur at the trauma centre, a transferring hospital, or in the ambulance (in limited cases). To assess our secondary objective, we will additionally consider the individual resuscitative interventions as separate exposures of interest.

### Data collection procedures

**Fig. 2:**
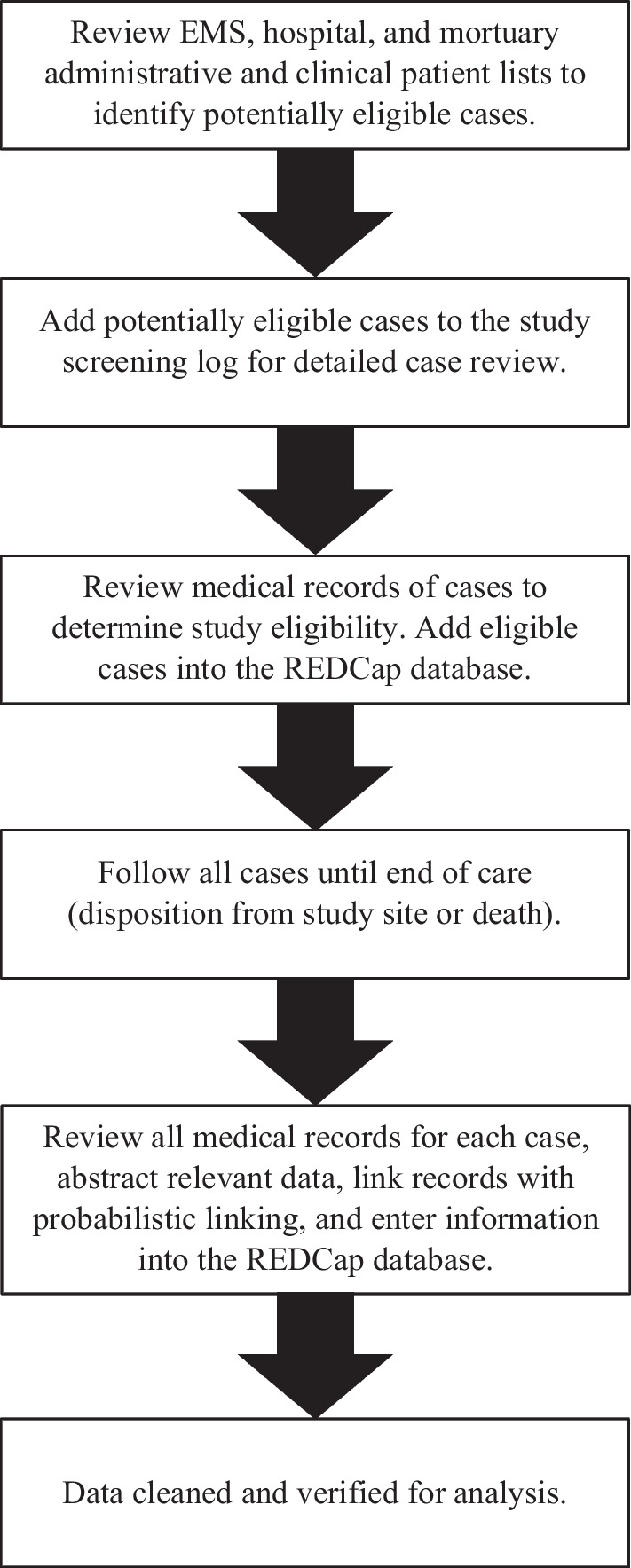
EpiC data collection process

Data will be collected on all eligible trauma patients using standardized clinical chart abstraction of prehospital, in-hospital, and forensic pathology records. Probabilistic linking will be used to confirm patient identities from each site prior to entry into the research database. Abstracted data are entered into a research electronic data capture tool—REDCap [55, 56]. A team of research data collectors have been trained to collect data in a standardized process with all variables defined in a data dictionary. The overall study procedure is summarized in Fig. 2.

## Statistical methods

Descriptive statistics will be estimated for all demographic, injury, facility, clinical, and systems process fields. Multivariable models will adjust for characteristics at the patient-level (e.g., age, sex, race, comorbidities, socio-economic status, injury mechanism, injury severity index) and provider-level (e.g., specialist trauma surgeon, general medical officer, advanced prehospital provider), as described in Table 2. Due to the small number of hospitals, site will be adjusted for as a fixed effect.

### Assessing association with mortality outcomes

Time-to-event analyses will be used to estimate the association between delivery and trauma-to-CRI time with mortality outcomes. This approach allows us to (1) systematically follow a representative cohort of injured individuals that are alive at the initial request for medical attention with moderate to high baseline risks, and (2) produce valid estimates of the timeliness and effectiveness of the urgent care initiated within the regional trauma system for these individuals [57]. Multivariable Cox proportional hazards models with a time-dependent covariate for CRI will be used to study the effect of receiving CRI during different time periods post-injury (e.g., 0–2, 2–12, > 12 h). We will assess model fit and the proportional hazards assumption. We will consider alternative model specifications as necessary, including a left truncated Cox proportional hazards model [58, 59], joint modelling of CRI and death [60–62], and estimating the survivor average causal effect (SACE) [63–66].

The secondary objective of assessing the effect of the delivery and timing of individual resuscitative interventions that occur during clinical course will be evaluated using similar models. Some interventions are injury-specific, and thus analysis will be performed in appropriate subgroups (e.g., by injury type and injury severity).

### Assessing association with morbidity outcomes

Analyses for morbidity outcomes will be conducted using multivariable generalized linear models (GLMs) with the appropriate link function chosen based on the outcome [67]. To account for potential informative censoring by the competing risk of death, we will use a weighted GLM with inverse probability of censoring weights (IPCW) [68, 69] and will compute robust standard errors. IPCW will be estimated from a logistic regression with the outcome of availability of morbidity information and predictors related to the morbidity and mortality (e.g., patient risk factors). In the weighted GLM, the association between delivery of CRI and morbidity outcomes will be assessed by including and testing the effect of a binary indicator for treatment. The effect of trauma-to-CRI time will be investigated using flexible, potentially non-linear (e.g., sigmoidal, exponential, piecewise) relationships.

For the secondary objective, we will examine the effect of individual resuscitative interventions on morbidity outcomes in treated patients using similar GLMs with intervention and time-to-CRI defined based on the specific treatment. Analysis will be performed in appropriate subgroups (e.g., injury type, injury severity) for injury-specific interventions.

### Missing data

Missingness of key measures will be minimized during the data collection process. The primary analysis will be a complete-case analysis that excludes observations that are missing key exposure, outcome, and predictor variables. Characteristics will be compared between those with and without complete data. Sensitivity analyses will be conducted using imputation methods for missing predictor values based on multiple imputation techniques [70].

### Power calculation

Based on a conservative estimate from the smallest hospital, we assume an average of 3,600 cases from each of the four hospitals in a 4-year period. Thus, we anticipate a total sample size of 14,400 cases over the study period. We assume that 30% of cases have an average trauma-to-CRI time within 2 h of injury and an average 7-day mortality of 10% for the primary mortality outcome. From a Cox proportional hazards regression, with 80% power (alpha = 0.05, two-sided) we can detect a hazard ratio of 0.85 for trauma-to-CRI time within the first 2 h versus not. For the morbidity outcomes, we present the power calculation for a continuous outcome (organ failure), and a binary outcome (wound infection). For the continuous outcome of organ failure, assuming the standard deviation of the SOFA score is 4 [71], with 80% power (alpha = 0.05) we can detect a mean decrease in score of 0.20 in those with trauma-to-CRI time within the first 2 h versus not, which corresponds to a standardized effect size of 0.05. For the binary outcome indicating whether a wound infection occurred, if 10% of cases with a trauma-to-CRI time within 2 h have a wound infection, with 80% power (alpha = 0.05, two-sided) we can detect an odds ratio of 0.84 for trauma-to-CRI time within 2 h versus not.

## Discussion

Globally, the burden of trauma remains high, particularly in LMICs and military populations. Timely delivery of quality trauma care is critical to improving trauma outcomes. Trauma patterns and determinants of outcomes need to be well characterized, epidemiologically and clinically, to strengthen clinical care, trauma policies, and system-level processes. In high-income countries and the U.S. military, the introduction of trauma databases and registries has led to a significant reduction in post-injury death and disability. Yet, trauma registries are non-existent in most LMICs and where they do exist, in both civilian and military settings, there are a myriad of opportunities to supplement or improve the quality of existing data [4, 72–74].

The EpiC study represents the first effort to build a standardized, multicenter database of trauma in South Africa, beginning in the Western Cape. This DOD funded study will focus on trauma outcomes of critically injured civilians, many experiencing prolonged trauma-to-CRI times, in a resource-limited, high-trauma mortality setting. In the EpiC study, critical information on injuries is obtained, including predisposing and epidemiologic factors of trauma risk, care, treatment history, and patient outcomes. Findings from EpiC will help fill critical gaps in the scientific body of literature relevant to strengthening care of persons in the Western Cape, South Africa, other LMICs, and in the U.S. military combat-wounded.

Data from EpiC will also provide rich clinical information, absent from existing Western Cape hospital databases, which can be used to evaluate the efficacy of interventions, including the effects of delays and minimum interventions needed. The prehospital data in EpiC will uniquely include systems-level data on transport times and transfer patterns. This information will shed light on operational and controversial practices, including the benefits of bypassing nearby facilities in favor of busy trauma centers in resource-limited and rural settings. At the institutional and trauma system administrative level, findings from EpiC will help guide resource allocation, provide surveillance data that can be used for trauma quality improvement programs, describe the trauma population and care received, and inform institutional and government reports aimed at improving care and patient outcomes. From the combat casualty perspective, the EpiC study can contribute data which are needed to help plan U.S. military missions, casualty management risk mitigation strategies, and understand the likely effects of injuries in the setting of prolonged times before reaching an advanced resuscitative/surgical team or definitive care center [75].

## Limitations

There are several limitations to this study design and methodology. First, the chart review and abstraction approach present challenges such as missing data and difficulty deciphering handwritten records. To mitigate this, the study will continuously monitor data completeness and provide ongoing feedback to data collectors. Second, EpiC data will be prone to selection bias as not all injured individuals seek medical attention, and because the study population is limited to those seeking care at specified facilities [76]. Additionally, EpiC does not collect information for individuals that die during non-EMS transport to the hospital, which will potentially exclude individuals with extremely severe injuries from our analysis and will limit the generalizability of results to this population. Further, EpiC began enrollment during the COVID-19 pandemic when trauma caseloads across South Africa and in the Western Cape Province were reduced by up to 50% [36], which can result in the reporting of rates that are lower compared to pre-COVID periods.

## Trial status

To date, the preparatory work (phase 1: September 30, 2019 to December 31, 2020) and pilot study (phase 2: January 1, 2021 to August 30, 2021) of the EpiC study have been completed and informed the structure and conduct of the main phase (phase 3: began September 1, 2021) that is ongoing. Trained research personnel are currently collecting study data at all EpiC study sites in the Western Cape Province and will conclude data collection in September, 2024.

## Data Availability

Not applicable as there are no data presented in this protocol.

## Abbreviations

CRI: Critical resuscitative intervention
EpiC: Epidemiology and outcomes of prolonged trauma care
FPS: Forensic pathology services
GEE: Generalized estimating equations
GLM: Generalized linear model
IPCW: Inverse probability censoring weights
LMICs: Low-and middle-income countries
